# Comparison of Methods for Isolating Exosomes from Plasma Subjects with Normal and High Fat Percentages †

**DOI:** 10.3390/life15030410

**Published:** 2025-03-06

**Authors:** Jacqueline Noboa-Velástegui, Juan Carlos León, Jorge Castro, Ana Fletes, Perla Madrigal, Iñaki Álvarez, Rosa Navarro

**Affiliations:** 1 Doctorado en Ciencias Biomédicas, Secretaría Académica, Centro Universitario de Ciencias de la Salud, Universidad de Guadalajara, Calle Sierra Mojada No. 950, Colonia Independencia, Guadalajara C.P. 44340, Mexico; jacqueline.noboa2713@alumnos.udg.mx; 2 Departamento de Biología Celular, Fisiología e Inmnunología, Institut de Biotecnologia i Biomedicina, Campus de Bellaterra, Bellatera, 08193 Barcelona, Spain; 3 Laboratorio de Microscopia Electrónica, Departamento de Patología, Instituto Nacional de Ciencias Médicas y Nutrición Salvador Zubirán, Vasco de Quiroga 15, Col. Sección XVI/Belisario Domínguez, Alcaldía Tlalpan C.P. 14080, Mexico; carlos.leonc@incmnsz.mx; 4 Departamento de Ciencias de la Salud y Ecología Humana, División de Desarrollo Regional, Centro Universitario de la Costa Sur, Autlán de Navarro C.P. 48900, Mexico; jorge.castro@academicos.udg.mx; 5 Instituto de Investigación en Enfermería y Salud Traslacional, Departamento de Enfermería Aplicada, Centro Universitario de Ciencias de la Salud, Universidad de Guadalajara, Guadalajara C.P. 44340, Mexico; lilia.fletes@academicos.udg.mx; 6 UDG-CA-701, Inmunometabolismo en Enfermedades Complejas y Envejecimiento, Departamento de Biología Molecular y Genómica, Centro Universitario de Ciencias de la Salud, Universidad de Guadalajara, Guadalajara C.P. 44100, Mexico; perla.madrigal@academicos.udg.mx

**Keywords:** plasma exosomes, exosome isolation methods, fat percentage

## Abstract

Adipose tissue is responsible for fat storage and is an important producer of extracellular vesicles (EVs). The biological content of exosomes, one kind of EV, provides information on aspects such as immunometabolic alterations. This study aimed to compare three plasma exosome isolation methods—using a commercial kit (CK), size exclusion chromatography (SEC), and differential centrifugation (DC)—and select the best one. Individuals categorized by normal and high body fat percentages were used. The DC and CK were proven to be the most advantageous out of the exosome isolation methods, so we suggest these methods for further protein and molecular analyses, respectively. Still, we emphasize the importance of selecting an appropriate methodology depending on the specific research objectives. At the same time, no statistical differences in exosome quality, morphology, total protein, or *microRNA* concentration were observed between individuals categorized by body fat percentage, so we suggest that the exosomal cargo varies in individuals with normal and high fat percentages.

## 1. Introduction

Exosomes, ranging in size from 50 to 150 nm, represent a subtype of extracellular vesicle (EV) due to their biogenesis and are found in several bodily fluids, including plasma, serum, urine, seminal fluid, tears, breast milk, the aqueous humor, and saliva, within diverse cell types, and in vitro cultures [1,2,3]. Exosomes work as carriers for biological material such as *DNA*, *mRNA*, *microRNA*, lipids, and proteins, thereby conveying information about the originating cells’ state and potentially influencing the function of recipient cells [4,5,6,7,8]. Adipose tissue (AT) is currently recognized as an essential source of circulating EVs, also known as adipocyte-derived extracellular vesicles (AdEVs), which function as a bridge between adipocytes and cells in the stromal fraction of AT, as well as cells from other systems [9,10]. AdEVs are filled with biological material that, in AT, plays a role in metabolic alterations, such as obesity, type 2 diabetes, and related illnesses, and maintain the body’s homeostasis [9,11,12].

Even though it is unclear how, in fat depot accumulation, AdEV cargo is involved in obesity-related diseases and how these signaling molecules vary, exosomes are an alternative communication pathway that can influence cellular and tissue functions by exerting stimulatory or inhibitory effects, such as promoting cell proliferation, inducing apoptosis, modulating cytokine production, and regulating immune responses [13]. Consequently, the significance of exosome content in developing AT dysfunction lies in their role as carriers of proteins that recruit macrophages to the AT, mainly TNFα and IL-6, which contribute to the onset of insulin resistance (IR) [4], and an imbalance in lipid metabolism can occur as exosomes carry enzymes associated with lipogenesis. Consequently, these enzymes may influence lipogenic activity in recipient cells [10]. On the other hand, this scenario occurs within AT and distant organs, as exosomes can damage endothelial cells, compromise blood vessels, exacerbate liver fibrogenesis, and promote polarization to the M1 macrophage phenotype [9]. The increase in fat mass, which translates into the accumulation of triglycerides within adipocytes, triggers persistent cellular apoptosis. This process creates a hypoxic microenvironment characterized by chronic low-grade inflammation, leading to dysregulation in the secretion of cytokines, adipokines, and other factors essential for AT homeostasis [12,14].

To ensure precision and reproducibility, it is essential to compare other methods with the gold standard of exosome isolation, typically represented by differential centrifugation (DC) [15]. Traditionally, exosome isolation involves sequential centrifugation steps, including low-speed centrifugation to remove cells and debris, followed by higher-speed ultracentrifugation to pellet exosomes. Density gradient centrifugation and ultrafiltration have also been employed to enhance purity. Recently, alternative methods have been incorporated to reduce time-consuming procedures, the use of specialized equipment, the number of samples, and the supplies required. Depending on the source of exosomes and research objectives, these methods include polymer precipitation, immunoaffinity capture, chromatography [16], size exclusion [17], and commercial kits [18,19]. Alternatively, to characterize exosomes, both optical and non-optical techniques are available, each chosen based on the specific information required for research purposes. Optical methods include dynamic light scattering (DLS), multi-angle light scattering, nanoparticle tracking analysis, flow cytometry, and surface plasmon resonance [20,21]. Non-optical approaches encompass scanning electron microscopy, transmission electron microscopy (TEM), cryogenic transmission electron microscopy (cryo-TEM), atomic force microscopy, Fourier transform infrared spectroscopy, and the labeling of exosome membrane proteins such as CD9, CD63, CD81, and CD82 [20,22,23,24,25].

Based on the eight exosome isolation methods recently updated in the MISEV2023 guidelines, differential centrifugation (DC) in combination with other approaches demonstrates a higher level of specificity, whereas precipitation methods alone offer superior recovery [3,26,27]; therefore, this study aimed to compare three plasma exosome isolation techniques: precipitation using a commercial kit (CK, Invitrogen^®^), size exclusion chromatography (SEC), and differential centrifugation (DC). The isolated exosomes were characterized using dynamic light scattering (DLS), cryo-TEM, TEM, and Western blot analysis targeting the CD9 and CD81 markers to facilitate subsequent protein and molecular biology assays.

## 2. Materials and Methods

### 2.1. Samples

Plasma Sample

Blood collection was approved by the *Comisión de Investigación y Ética del Antiguo Hospital Civil de Guadalajara “Fray Antonio Alcalde” O.P.D* Guadalajara, México. HCG/CEI-0835/22, N°. 130/22 after 118 participants aged 20 to 59 (71 women and 47 men) provided their written informed consent and were classified by fat percentage. A high fat percentage was considered to be more than 25% in men and 35% in women. We included 59 individuals with normal and 59 with high fat percentages. Ten milliliters of blood were obtained in EDTA-coated tubes and allowed to sit at room temperature for 30 min. The whole blood was centrifuged at 3000× *g* for 15 min at room temperature to separate plasma. The individual plasma samples were stored at 4 °C until the experiments. The clinical and anthropometric characteristics of the study subjects are described in Appendix A.

### 2.2. Exosome Isolation and Characterization

#### 2.2.1. Isolation by Differential Centrifugation (DC)

Two mL of blood plasma was used, and all centrifugations were performed at 4 °C. We started with 300× *g* for 10 min (Heraeus Megafuge 2.0 R) and discarded the pellet; 2000× *g* for 20 min (Heraeus Megafuge 2.0 R) and discarded the pellet; 10,000× *g* for 20 min (Beckman Coulter JA-14 rotor, Brea, CA, USA; 250 mL NALGENE^®^ bottles, Asheville, NC, USA) and discarded the pellet; 100,000 g for 70 min (Beckman Colter T70i rotor; 26.3 mL Beckman centrifuge tubes), discarding the supernatant and resuspending the pellet with 500 μL of 1X PBS; and 100,000 g for 70 min (150K rpm Thermo Sorvall Discovery M150 SE floor micro-ultracentrifuge; S150-AT rotor; 1 mL Eppendorf tubes PP) and resuspended the pellet in 200 μL of 1X PBS [28].

#### 2.2.2. Isolation by Size Exclusion Chromatography (SEC)

One mL of blood plasma at room temperature was used. Broadly speaking, we centrifugated it at 2000× *g* for 10 min and used the supernatant for a second centrifugation at 10,000× *g* for 30 min at 10 °C; the supernatant was filtrated through a 0.22 μm filter (CORNING; 45 mm diameter). Finally, we passed the sample through each column (Pkg of 50 #7321010 Econo-Pac^®^ chromatography columns with Sepharose™ CL-2B (BioRad, Barcelona, Sapin)) with 1X PBS at room temperature filtrated through a 0.22 μm filter. Then, 200 μL of 15 fractions was collected [17,29].

#### 2.2.3. Isolation by Precipitation with a Commercial Kit (CK)

A total exosome isolation (from plasma) kit (Invitrogen Cat. No. 4404450 (Vilnius, Lithuania)) was used according to the manufacturer’s recommendations. All centrifugations were performed at room temperature. Starting with 500 μL of blood plasma, followed by centrifugation at 10,000× *g* for 20 min, we added 0.5 volumes of PBS 1X to the supernatant, mixed it by vortexing, added 0.2 volumes of exosome precipitation reagent, and pipetted it up and down. This mix was incubated for 10 min at room temperature, then centrifugated at 10,000× *g* for 5 min, and the supernatant was discarded. Finally, the pellet was resuspended in 50 μL of PBS 1X.

#### 2.2.4. Characterization by Dynamic Light Scattering (DLS)

The diameters of the EVs were analyzed by DLS using 500 μL of sample dissolved in 10 mM NaCl [30]. The equipment was set up at 25 °C for 60 min (Antor Paar. Litesizer DLS 700, Malverne Zetasizer Software v7.13 PSS0012-39. Gratz, Austria).

#### 2.2.5. Characterization by Cryo-TEM and TEM

For TEM, negative staining was performed with a 1:1 proportion of isolated exosomes and glutaraldehyde; 6 μL of this mix was washed and collocated in the carbon mesh [31] for transmission electron microscopy (FEI Technology, Model Tecnai Spirit BioTwin, software FEI TIA v4.15), and 3 μL of isolated exosomes was used for cryo-TEM (Jeol JM-2011, Tokyo, Japan) [32].

#### 2.2.6. Characterization by Western Blot

Starting with an exosome lysis buffer (25 mM Tris-HCl +120 mM NaCl + 1% Triton-X100 + complete Mini, EDTA-Free; Merck, Darmstadt, Germany), we added 20 μL of it to 150 μL of the sample in ice-cold conditions, then incubated it with rotational agitation at 4 °C for 1 h, and then centrifugated it at 15,000× *g* for 15 min at 4 °C. The supernatant was measured with a spectrophotometer (NanoDrop 1000 UV visible spectrophotometer, Thermo Scientific (San Diego, CA, USA)). We charged 5 μg of total protein on 14% acrylamide gel. Exosome markers were quantified using mouse monoclonal CD9 (1:1000; antibodies.com; Anti-CD9 (MEM-61) (A86089)), CD81 (1:1000; antibodies.com; Anti-CD81 (M38) (A86719)), and vinculin (1:500 Invitrogen Ref. MA5-11690 Barcelona, Spain). We used anti-mouse HRP (1:5000; Abcam; NA931V ECL Anti-mouse IgG) and ECL (BioRad Clarity Western ECL substrate 500 mL; #1705061. Barcelona, Spain) in a spectrophotometer (Li-cor, Odyssey XF, Lincoln, USA. LI-COR Acquisition Software v2.2).

### 2.3. MicroRNA Isolation

Trizol reagent was used to isolate *microRNA*—50 µL of isolated exosome was lysed in 200 µL Trizol + 1 µL (1 µg/µL glycogen, *RNA* grade; Thermo Scientific #R0551). Subsequently, 40 µL of chloroform was used for phase separation, and 100% isopropanol was used for *microRNA* precipitation. Finally, *microRNA* was eluted in 30 µL of RNase-free water after being washed twice in 75% ethanol. The *microRNA* concentration was assessed using Qubit^®^ 4.0.

### 2.4. Statistical and Image Analyses

For each method, one-way ANOVA, post hoc Tukey tests, and the Mann–Whitney *U* test (mean ± SD) were used to compare the total protein and *microRNA* concentration differences between average and high fat percentages. *p* < 0.05 was considered significant. GraphPad Prism version 8.4.0 for macOS was used for data analysis and graphing. ImageJ2 version 2.1.4.0/1.54f was used for image analyses.

## 3. Results

### 3.1. The Exosome Isolation Methods Showed Equal Performances in Total Protein and microRNA Concentration, While an Inverse Pattern Was Observed Among Individuals with High Fat Mass Contents

**Figure 1:**
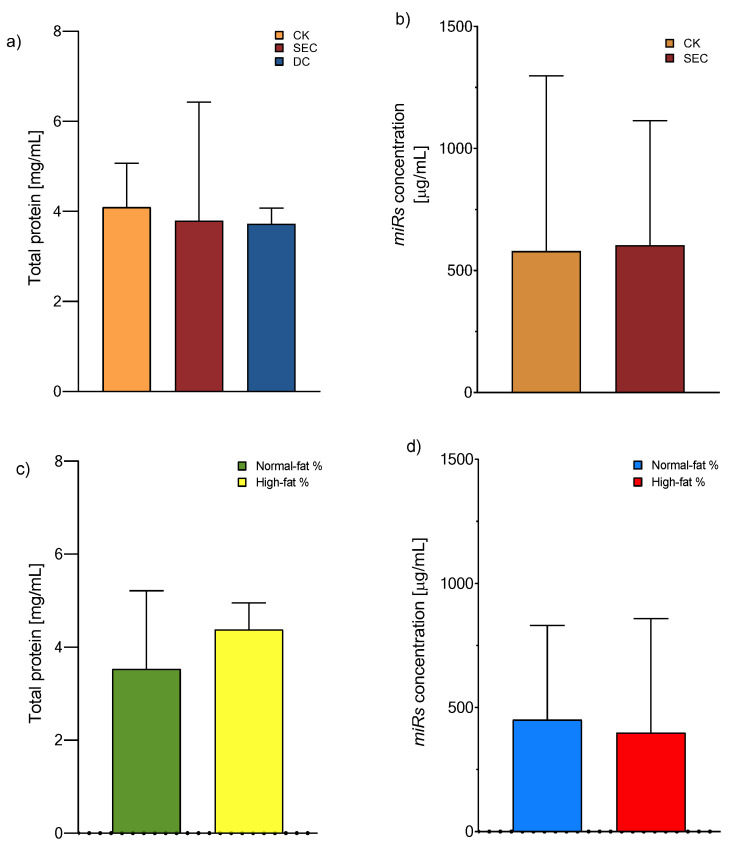
Exosome total protein and *microRNA* concentration. (**a**) Protein concentration means of 4.09, 3.97, and 3.97 mg/mL for the CK, SEC, and DC, respectively (one-way ANOVA and post hoc Tukey tests), and (**b**) microRNA concentration means of 579 and 603 mg/mL for the CK and SEC, respectively (Mann–Whitney U test). Both were measured from exosomes, *n* = 118, with no significance. (**c**) Total protein concentration and (**d**) total microRNA concentration (Qubit assay kits, Invitrogen™) from plasma exosomes isolated by a commercial kit (Mann–Whitney U test, no significance). Abbreviations: CK: commercial kit; SEC: size exclusion chromatography; and DC: differential centrifugation.

The exosomes were isolated according to the manufacturer’s recommended instructions for the CK, whereas DC and SEC were utilized for classical ultracentrifugation and column fraction separation, respectively. The exosome yield was determined by the total protein concentration using a Qubit Protein Assay (Invitrogen™, Eugene, OR, USA). We observed that the precipitation-based total exosome isolation kit (Invitrogen) had the maximum yield, followed by SEC and DC (Figure 1a). After these results, we chose a CK and SEC for isolating the *microRNA* from the exosome with equal performances (Figure 1b). On the other hand, there is a tendency for the concentration of total proteins or miRNAs to increase, although the values did not reach statistical significance (Figure 1c,d).

### 3.2. The Morphology and Quality of the Three Exosome Isolation Methods Were as Expected, with Inconsistencies in Purity

**Figure 2:**
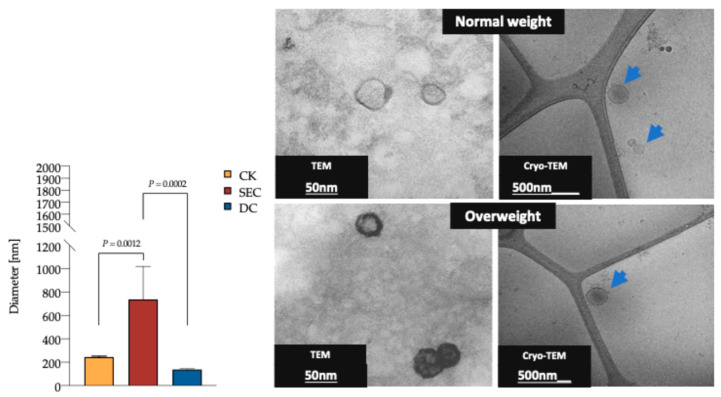
DLS of plasma exosomes isolated by the CK, SEC, and DC, as well as TEM and cryo-TEM exosome photomicrography. The exosomes’ average diameter was 235.1 nm, 736.9 nm, and 138.2 nm for the CK, SEC, and DC, respectively (*n* = 15 for each method, one-way ANOVA, and post hoc Tukey tests; *p* < 0.05). TEM of exosomes isolated by the CK (Invitrogen Cat. No. 4404450). Diameter of 53.9 nm, scale of 50 nm. Cryo-TEM of exosomes isolated by SEC. Diameter of 191.7 (blue arrow) nm, scale of 500 nm. Exosomes from plasma samples of individuals were classified by fat percentage (59 for each group). Abbreviations: DLS: dynamic light scattering; CK: commercial kit; SEC: size exclusion chromatography; and TEM: transmission electron microscopy.

The DLS analysis revealed that the vesicle diameter measured in the CK and DC was consistent with the expected exosome size (Figure 2); however, the purity of the vesicle subpopulation varied between the CK and DC compared to SEC. The TEM and cryo-TEM analyses confirmed that the morphology and overall quality of exosomes were consistent across methods. Additionally, no morphological differences were observed between exosomes from individuals with normal and high fat percentages (Figure 2).

### 3.3. CD9 and CD81 Do Not Differ Between Normal- and High-Fat-Percentage Individuals in SEC Fractions

**Figure 3:**
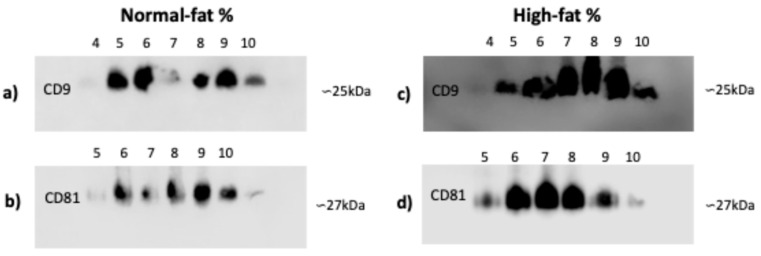
CD9 and CD81 markers of exosomes isolated by SEC. (**a**,**c**) CD9 marker: the presence of exosomes of the exclusion fractions from 4 to 10. (**b**,**d**) CD81 marker: the presence of exosomes of the exclusion fractions from 5 to 10. Exosomes were isolated from plasma samples of individuals that were classified by fat percentage: (**a**–**d**) high (59 for each group). Abbreviation: SEC: size exclusion chromatography.

Since the integrity and quality of microscopy images and total protein did not differ, we chose an exosome isolation technique that was more cost-effective and equipment-efficient for identifying CD9 and CD81 in plasma exosomes from individuals with normal and high fat percentages. In individuals with normal and high fat percentages, there are no shifts in CD9 marker fractions from four to ten (Figure 3a,c) or in CD81 fractions from five to ten (Figure 3b,d).

Based on our experience, we can establish some characteristics of these three exosome isolation methods, as shown in Table 1 below.

## 4. Discussion

It is estimated that the prevalence of obesity and overweight will reach 1.7 billion people by 2030 [33], so understanding the mechanism by which AT acts under these conditions is critical to preventing, treating, and reversing the loss of homeostasis within this tissue. The adipocyte-derived extracellular vesicles play an essential role in developing obesity-associated diseases, like cancer [34], among others; as suggested by Delgadillo et al., AdEV size can be related to liver and systemic IR [35].

Currently, the choice of method with which to isolate exosomes is wide-ranging, depending on the application of these vesicles. Ultracentrifugation, preceded by low-speed centrifugation, is the gold-standard technique [36]. Still, the equipment and accessories for it are not available in all laboratories. In the present study, we gauged the material and equipment price, time consumption, vesicle yield, purity subpopulation, protein, and *microRNA* concentration of three isolation methods to define the one most suitable for further analyses. The results were as follows: (a) the CK presented the highest total protein concentration in contrast with the other two techniques; (b) there was a similarity in *microRNA* concentration between the exosomes isolated with the CK and SEC; and (c) there was a likeness in exosome total protein and *microRNA* concentration between normal- and high-fat-percentage individuals.

Even though total protein quantification is a suitable method for estimating the concentration of exosomes, it cannot distinguish between contaminating proteins, like albumin, that are not associated with them [27]; therefore, between the three methods, the commercial kit is the most reproducible, as mentioned by Caradec et al. [37]. After comparing the concentrations of the total protein content of the exosomes by using the three methods, we decided to evaluate normal and high percentages of fat and the total *microRNA* by using the most straightforward method, which is less time-consuming and allows for the analysis of a more significant number of samples. Precipitation by a CK is an excellent alternative to a subsequent analysis of nucleic acid, even if we could not find a difference between the three methods, as Mohammad et al. also determined when comparing a commercial kit to ultracentrifugation [38]. In this context, without a difference between individuals with normal and high fat percentages, considering Wang et al.’s evaluation of the exosome proteome in obese and non-obese individuals with T2DM [39], we can propose that the exosomal cargo is linked to metabolic-associated disorders rather than fat content [40,41].

Many techniques have been used for exosome characterization [42], depending on the information that researchers require. In our case, we chose two that can be used for quality (protein marker, shape, and size) analyses: DLS, electron microscopy (TEM and cryo-TEM), and Western blot (CD9 and CD81 markers). DLS allowed for the characterization of plasma exosome subpopulations according to their diameters, as shown by the CK and DC methods. In contrast, SEC appears to yield a higher presence of aggregated exosomes and other plasma particles of similar sizes, probably due to the intrinsic nature of its isolation mechanism [43]. Electron microscopy enables the imaging of single exosomes, visualizing their size and morphology. Exosomes isolated by the CK and DC demonstrated the presence of fewer microvesicles, while SEC presented a considerable microvesicle population, as mentioned by Davidson et al. [44].

We chose two tetraspanins to verify that we isolated microvesicles compatible with the exosomes (CD9 and CD81). We analyzed normal- and high-fat-percentage individuals, in whom there was a difference in the delay of fractions between markers but not in fat condition. This pattern was shown in carcinoma exosomes [45] and circulating exosomes [46] isolated by SEC. Following our results, Sharif et al. mentioned that SEC is a suitable method for further nucleic acid analyses, by which they also found a distinct population of EVs [47]. Our decision to use the three exosome isolation methods and exosome characterization is supported by the latest update of the International Society for Extracellular Vesicles (MISEV2023 [3]), as well as the advantages and disadvantages found according to our results (Table 1).

After evaluating the three exosome isolation techniques, we identified specific limitations linked with each method. In the case of differential centrifugation (DC), a major drawback is the requirement for access to ultracentrifugation or miniultracentrifugation equipment, which is not available in all laboratories. Similarly, for size exclusion chromatography (SEC), noteworthy challenges include the extended time required for sample processing and variability between protocols, particularly regarding the initial sample volume and the volume collected from each fraction.

Exosomes have garnered significant interest due to their potential as biomarkers, as they transport proteins and *microRNAs* capable of predicting the diseases associated with obesity. Furthermore, they enable the evaluation of the functional status of adipose tissue and are emerging as promising therapeutic vesicles for restoring homeostasis in this tissue. Notably, by conducting a comprehensive immunometabolic assessment of an individual, exosome-based therapies could be tailored, paving the way for personalized medical approaches.

## 5. Conclusions

We highlight the importance of selecting the most suitable exosome isolation method based on specific research objectives. For studies prioritizing exosome integrity, we recommend using SEC for plasma samples, as it yields a higher concentration of intact exosomes. However, if the focus is on achieving greater purity in EV subpopulations, DC or a commercial isolation kit are more appropriate methodologies. We suggest the DC and CK methods for further protein and molecular analyses, respectively.

Our results indicate that circulating exosomes do not differ in quantity and quality between individuals with normal and high body fat percentages; however, their compositions do vary. We recommend further investigations into the molecular cargo of circulating exosomes, as it may serve as an indicator of adipose tissue status and could help elucidate these differences.

## Figures and Tables

**Table 1:** Comparison between three methods for exosome isolation.

Isolation Method	Advantage	Disadvantage
Commercial kit (CK)	Fast procedure Many samples can be tested at the same time (centrifugation rotor tube capacity) Small volumes of samples Does not require expensive or complicated equipment Easy technique High yield Exosome integrity is maintained	Relative price Kit stability Does not have a high purity (for further proteomic analysis)
Size exclusion chromatography (SEC)	Economical material Non-destructive High yield Exosome integrity is maintained	More time-consuming procedure Sample quantity is limited in order to test them at the same time High volumes of sample
Differential centrifugation (DC)	Purity (exosome size) Many samples can be tested at the same time (ultracentrifugation rotor tube capacity) Small volumes of samples (miniultracentrifuge) High yield	More time-consuming procedure High volumes of sample (conventional ultracentrifuge) Expensive equipment Pressure damages the exosomes’ integrity Induces aggregation of exosomes

## Data Availability

Information is available upon reasonable request to the corresponding author.

## Appendix A

Clinical and anthropometric characteristics of the study subjects.

**Table:**

Groups	Normal Fat Percentage	High Fat Percentage	*p*-Value
n	59	59	
Sex (Men–Women)	26:33	21:38	
Age	32 ± 9	38 ± 10	
Lipid Profile			
TC (mg/dL)	173.4 ± 32.8	191.4 ± 44.5	0.01 *
Triglycerides (mg/dL)	95.6 ± 49.5	199.7 ± 202.3	<0.0001 ****
Insulin Resistance Status			
Fasting glucose (mg/dL)	75.6 ± 17.3	88.7 ± 42.7	0.01 *
Fasting insulin (uUI/mL)	12.5 ± 9.8	21.9 ± 11.5	<0.0001 ****
Evaluation of Body Adiposity Status			
BMI (kg/m^2^)	24.5 ± 2.9	34.0 ± 5.4	<0.0001 ****
Fat %	24.5 ± 6.4	38.8 ± 7.7	<0.0001 ****
AVI (cm^2^)	14.2 ± 6.3	22.8 ± 7.7	<0.0001 ****
VAI	1.4 ± 3.3	0.2 ± 0.1	<0.0001 ****
WC	81.6 ± 14.1	103.2 ± 21.1	<0.0001 ****

Mann–Whitney *U* test (mean ± SD). Significance: * *p* < 0.05, **** *p* < 0.0001. Abbreviations: TC, total cholesterol; BMI, body mass index; BAI, body adiposity index; AVI, abdominal volume index; VAI, visceral adiposity index; and WC, waist circumference.
